# Estimating Health Risks to Children Associated with Recreational Play on Oil Spill-Contaminated Beaches

**DOI:** 10.3390/ijerph18010126

**Published:** 2020-12-27

**Authors:** Tanu Altomare, Patrick M. Tarwater, Alesia C. Ferguson, Helena M. Solo-Gabriele, Kristina D. Mena

**Affiliations:** 1 Department of Epidemiology, Human Genetics & Environmental Sciences, UTHealth Houston School of Public Health, Houston, TX 77030, USA; tkaur825@gmail.com; 2 Department of Epidemiology, Johns Hopkins Bloomberg School of Public Health, Baltimore, MD 21202, USA; tarwater@jhu.edu; 3 Built Environment Department, North Carolina A&T State University, Greensboro, NC 27411, USA; acferguson@ncat.edu; 4 Department of Civil, Architectural, and Environmental Engineering, University of Miami, Coral Gables, FL 33146, USA; hmsolo@miami.edu

**Keywords:** risk assessment, children’s health, oil spills

## Abstract

The human health impact from exposure to contaminated shorelines following an oil spill event has been investigated to some extent. However, the health risks to children have largely been characterized through the use of surveys and extrapolation from adult health outcomes. There is limited information on children’s behaviors during beach play requiring assumptions made based on observations from play activities in home settings. The Beach Exposure and Child Health Study (BEACHES) quantified specific beach activities that can be used to inform human health risk assessments of children playing on beaches impacted by oil spills. The results of this study characterize children’s risk of cancer from exposure to oil spill chemicals by incorporating exposure-related information collected from the BEACHES study and by assuming oral, dermal, and inhalation exposure routes. Point risk estimates are compared with a previous, similar study that applied default exposure parameter values obtained from the published literature. The point risk estimates informed by BEACHES data are one order of magnitude lower compared with the previous risk assessment, with dermal exposures the overall risk driver in both. Additional Monte Carlo simulations evaluating the BEACHES data provide ranges of health risks with the highest estimates associated with dermal and oral exposure routes.

## 1. Introduction

In 2010, the British Petroleum-operated Deepwater Horizon (DWH) drilling rig, located about 60 km offshore from the Louisiana coast, exploded resulting in the release of over three million barrels of oil along the shorelines of Texas, Louisiana, Mississippi, Alabama, and Florida [1]. The DWH oil spill is considered the largest marine oil spill in history and has led to harmful effects on the economy, ecosystem, and health of communities living along the Northeastern Gulf of Mexico [2,3,4]. The composition of crude oil contains several types of alkanes, aromatic hydrocarbons, metals, and inorganic compounds [5]. Additional chemical by-products are created when crude oil comes into contact and reacts with water and air [6]. A majority of the existing literature on the impact of DWH on human health focuses on the health of first responders [7,8,9], especially in regard to exposure to dispersants, as well as physical [10] and mental health [11] outcomes of adults living in affected areas. Children, however, may be particularly vulnerable to physical adverse health consequences from subsequent exposures to environmental contaminants associated with oil spills.

Although there is existing literature characterizing the health risks to adults from exposure to oil spill chemicals, analyses specific to children’s health are limited [12]. Children’s age-specific behaviors—such as mouthing, crawling, and inconsistent hygiene—can put them at greater risk of exposure to environmental contaminants [13,14] and, therefore, health consequences. Children’s greater frequency of hand-to-mouth contact also puts them at higher risk of exposure from non-dietary ingestion routes [15]. There have been studies in residential settings evaluating the dynamics of children’s behavior in the environment and subsequent exposures to hazards [16,17], and some investigations characterizing children’s exposure-related activities in the beach environment [18,19,20,21,22,23,24]. Shoaf et al. [19] evaluated the extent of dermal exposure by children to sediment in a tide flat by collecting information from parents regarding children’s play behavior as well as measuring adherence of sediment to children’s skin. More recently, Ferguson et al. [21] identified the different ways children play on the beach and how this impacts environmental exposures. Information from such studies can be used to predict children’s health risks by defining exposure-related parameters.

The human health risk assessment framework for chemical exposures was first developed in the late 1970s by the National Research Council [25] and initially applied to determine if an unknown environmental chemical was harmful to humans. The framework is now used to evaluate the extent of human health outcomes associated with exposure and to address a range of hazards and environmental sources. Risk assessment methods have been applied to address scenarios associated with natural disaster preparedness and damage mitigation [26], as well as to evaluate responses to earthquakes, hurricanes, monsoons, floods, and tornadoes [27,28,29]. Because of its flexibility, the risk assessment framework can be applied to address different exposure scenarios and sub-populations, including children. Population-specific exposure variables, such as inhalation rate and exposure frequency, can be assumed to predict the chance of adverse health outcomes from contaminants in specific environments. In the beach environment, children likely spend more time exposed to sand—especially in the intertidal zone—where contaminants may accumulate [30,31].

The objective of this study was to conduct a human health risk assessment utilizing child-specific data collected from the Beach Exposure and Child Health Study (BEACHES) to predict the probability of cancer among children under the age of seven years exposed to oil spill chemicals in the beach environment. Predicted risk estimates were compared to results generated from a previous, similar risk assessment that assumed default risk model parameter values available in the literature [32]. The BEACHES study was conducted in May and July 2018 in Miami, Florida and Galveston, Texas (respectively), where exposure factors impacting children’s health risks were collected or observed rather than assuming default values. This is important as exposure-related parameters impact human health risk assessment output making it critical to consider children’s play behavior when characterizing their environmental exposures. Unlike the previous study [32], this present study also included Monte Carlo simulations to address the variability intrinsic to the exposure assessment component. Chemical information was obtained from existing chemical concentration data from shoreline sampling following the DWH oil spill in 2010.

## 2. Methods

This study utilized the components of the National Research Council (NRC) risk assessment framework: hazard identification, exposure assessment, dose–response assessment, and risk characterization [25]. In the hazard identification step, the agent of interest was evaluated for its ability to cause adverse consequences based on information found in the peer-reviewed literature. Information on associated illnesses due to exposure was described and any data regarding the percent of symptomatic individuals, as well as acute and chronic health outcomes, were considered. The exposure assessment component identified the different environmental sources of the hazard and recognized the various ways the hazard may be transmitted. This may result in the identification of new exposure pathways or a combination of possible transmission routes. For many environmental agents, toxicity data are available with dose–response relationships established in the published literature to be used in health risk assessments. Finally, the information learned in the assessment is integrated so that health risks can be estimated qualitatively and/or quantitatively (risk characterization) [25].

Although many chemicals are part of an oil spill profile, benzo[b]fluoranthene was chosen as the chemical hazard to evaluate in this risk assessment due to existing data that inform toxicity-related variables in a risk assessment (Table 1). In addition, concentrations for benzo[b]fluoranthene were reported from sampling conducted by the United States Environmental Protection Agency (USEPA) during and immediately following the DWH oil spill (28 April 2010 to 6 October 2010) [32]. The following risk equations were used to generate point estimates and ranges of cancer risk due to exposure to beach sediment, weathered oil, or tar. Equation (1) defines risk as a function of dose and cancer slope factor with the latter specific to exposure route (oral, dermal, or inhalation):

(1) Risk=Dose×Slope factor

The slope factor was defined as the estimated cancer risk per mg per kg body weight per day. The slope factor approximated a 95% confidence limit on the increased cancer risk from a lifetime of exposure to a hazard [33]. Slope factors were generated for suspected carcinogens by extrapolating low doses using high-concentration dose–response assays in animal models [34].

Dose estimations are dependent on the route of exposure and can be combined for aggregate exposure estimates [35]. For non-dietary ingestion (oral) exposure to beach sediment, weathered oil, or tar, the following equation was used:

(2) $${\mathrm{Dose}}_{\left(\mathrm{oral}\right)}=\frac{C\times {\mathrm{IR}}_{s}\times \mathrm{RBA}\times \mathrm{EF}\times \mathrm{CF}}{\mathrm{BW}}$$

where C = concentration (mg/kg), IR_s_ = soil intake rate (mg/kg), RBA = relative bioavailability factor (unitless), EF = exposure factor (unitless), CF = conversion factor (mg/kg), and BW = body weight (kg). The exposure factor was defined as:

(3) $$\mathrm{Exposure}\;\mathrm{factor}\;\left(\mathrm{EF}\right)=\frac{F\times \mathrm{ED}}{\mathrm{AT}}$$

where F = frequency of exposure (days/year), ED = exposure duration (years), and AT = averaging time (days). For dermal exposure, dose was calculated as:

(4) $${\mathrm{Dose}}_{\left(\mathrm{dermal}\right)}=\frac{C\times \mathrm{SA}\times \mathrm{AF}\times \mathrm{ABS}\times \mathrm{EF}\times \mathrm{CF}}{\mathrm{BW}}$$

where C = concentration (mg/kg), SA = skin surface area (cm^2^/event), AF = adherence factor for beach sand (mg/cm^2^), ABS = absorption factor (unitless), EF = exposure factor (unitless), CF = conversion factor (mg/kg), and BW = body weight (kg).

For inhalation exposure, it was assumed that exposure was due to the inhalation of suspended particulates since benzo[b]fluoranthene concentrations in the air were not available. The dose, as related to inhalation exposure, was defined as:

(5) $${\mathrm{Dose}}_{\left(\mathrm{inhalation}\right)}=\frac{C\times \frac{1}{\mathrm{PEF}}\times {\mathrm{IR}}_{a}\times \mathrm{ET}\times \mathrm{EF}}{\mathrm{BW}}$$

where C = concentration (mg/kg), PEF = soil-to-air particulate emission factor (m^3^/kg), IR_a_ = inhalation rate (m^3^/day), ET = exposure time (hours/day), EF = exposure factor (unitless), and BW = body weight (kg).

Monte Carlo analysis is a method used in human health risk assessments that involves computer simulations to consider different probability distributions in a risk model [36,37,38]. Using a specified range, each simulation randomly selects a value for each variable of a risk equation. Assuming these variables are independent of each other, the simulation calculates the resultant risk value. This process is then repeated for many iterations with the final result representing the range of outputs from each iteration. Monte Carlo simulations were conducted in this study using Microsoft Excel, Oracle^®^ Crystal Ball, assumed triangular distributions for the three BEACHES datasets, and the chemical concentration values for benzo[b]fluoranthene. Minimum and maximum values were taken from each dataset; median values were used in place of the likeliest value (Table 2). For chemical concentration, weathered oil represented the upper limit that could exist in sediment following shoreline oiling [32]. The exposure factor was calculated from the frequency of exposure, exposure duration, and averaging time. The Monte Carlo simulations ran for 1000 iterations and risk ranges were generated for oral, dermal, and inhalation exposures. Mean 2.5% and 97.5% cancer risk values for each exposure route were determined.

## 3. Results

Benzo[b]fluoranthene is an oil spill chemical classified as a polycyclic aromatic hydrocarbon; specifically, a five-ring structure generated from incomplete combustion of organic matter, such as coal and petroleum [39]. It is categorized as a probable human carcinogen based on information from animal studies; however, insufficient evidence exists for human exposures [40]. Animal bioassays involving benzo[b]fluoranthene demonstrated tumors from oral, dermal, and inhalation transmission routes. Exposure concentration values used in this assessment were determined for sediment, tar, and weathered oil sources [32]. The slope factors for oral, dermal, and inhalation assumed were obtained from the Center for Environmental and Human Toxicology (CEHT) [41] (Table 1).

The BEACHES study provided data to the following beach and population-specific parameters: frequency of exposure, exposure factor, body weight, and skin surface area. The remaining variables needed to complete the risk assessment were consistent with those used in Black et al. [32] (Table 3). Body skin surface area was calculated using established formulas [22,42,43] based upon a child’s height and weight. The assumed height and weight estimations represented averages obtained from demographic and survey information collected on behalf of 122 children who participated in the BEACHES study.

For the BEACHES study, participating children ranged in age from 0 to 6 years. In the previous analysis by Black et al. [32], parameters associated with children (body weight, soil intake rate, and inhalation rate) corresponded to children between the ages of 2 to 10 years (Table 3). Average values for frequency of exposure (3 vs. 12 days per year visiting the beach environment), exposure factors, and skin surface areas (12,582 cm^2^ vs. 11,350 cm^2^) generated from the BEACHES study were lower compared with benchmark values used in the previous risk assessment [32], whereas average body weight was recorded as higher (34.8 kg vs. 25.4 kg) in the BEACHES study.

Cancer risks for benzo[b]fluoranthene estimated from the BEACHES study data were one order of magnitude lower in total aggregate risk compared with risks reported by Black et al. [32] for both oral (5.73 × 10^−8^ vs. 2.78 × 10^−7^) and dermal (5.19 × 10^−7^ vs. 2.01 × 10^−6^) exposure routes. However, risk estimates from inhalation exposure were one order higher in the BEACHES analysis (1.13 × 10^−12^ vs. 5.80 × 10^−13^). Overall, risk estimates associated with inhalation exposure were several orders of magnitude lower compared with oral and dermal risk estimates (Table 4).

When computing the mean 2.5% and 97.5% cancer risk estimates for each exposure route, the mean cancer risks from the Monte Carlo analysis for oral and dermal transmission were one order of magnitude higher compared with their corresponding point estimate risk values (2.63 × 10^−7^ vs. 5.73 × 10^−8^ for oral exposure and 2.82 × 10^−6^ vs. 5.19 × 10^−7^ for dermal exposure) (Table 5). The mean risk estimate for inhalation exposure was six orders of magnitude lower compared with the inhalation point estimate (5.13 × 10^−18^ vs. 1.13 × 10^−12^).

## 4. Discussion

Population-based and behavioral variables (body weight, skin surface area, and frequency of exposure) that can be incorporated in risk assessments were generated from the BEACHES study. Averages for each variable were used to generate point risk estimates for oral, dermal, and inhalation exposure routes to beach sediment, weathered oil, and tarballs. Risk estimates generated using data collected from the BEACHES study were slightly lower than a previous risk assessment conducted by Black et al. [32]; however, mean risks estimated through a Monte Carlo simulation using BEACHES data were comparable to the Black et al. results. The lower risk estimates are due to the difference in defined risk model parameters determined from data collected during the BEACHES study for both skin surface area (12,582 cm^2^ vs. 11,350 cm^2^) and frequency of exposure (3 vs. 12 days per year). Further, bodyweight from the BEACHES study was observed to be higher compared with the weight benchmarks used in the Black et al. [32] assessment (34.8 kg vs. 25.4 kg). These differences explain the resulting lower risk estimates, especially regarding body weight, which is found in the denominator of all three risk equations.

For the point risk estimates and Monte Carlo analysis from the current study, overall risk values were highest in dermal exposure routes and lowest in inhalation exposure routes. Further, skin surface area exposure was reported to be higher for the BEACHES study compared with the Black et al. study [32], resulting in dermal exposure likely being a driver of overall risk. In context, children playing at beaches might be at greater risk from prolonged skin contact with contaminated sand and water, whereas the risk of exposure via the inhalation pathway might not be of greatest concern. This risk process assumed a single load of contact between the skin and the chemical. Children playing at the beach may experience multiple loading events, depending on how often they might enter the water or wash the sand off their skin, where actual loading on the skin influences uptake rates for dose estimates. Future assessments using data from video observations from the BEACHES study regarding sequential loading events will refine the exposure assessment and likely impact risk estimations associated with dermal exposure. Additionally, the equation used to calculate dose from dermal exposure incorporates adherence and absorption factors, but does not consider sand particle size distribution or whether sand is wet or dry—both factors impact the amount of time contaminated sand is in contact with skin [23,44].

For these risk assessments, it was assumed that children playing in the beach environment would be exposed to the same concentrations of benzo[b]fluoranthene consistently in all locations of the beach environment. However, the concentration of this oil spill chemical will vary in different beach areas such as within the intertidal zone, water, and sand dunes [45]. Moreover, it was assumed in this risk assessment that children would be exposed to the same chemical concentrations via the oral, dermal, and inhalation routes regardless of the microenvironment within the beach areas (such as dune and back trough). Depending on the location of play, children might experience higher exposure to some chemicals via certain routes and experience little-to-no exposure through other pathways. Although benzo[b]fluoranthene is commonly found in air impacted by cigarette smoke, soot, and gasoline exhaust; the concentrations used in this assessment are not from gaseous-phase measurements but assume solid-phase particulates suspended in the air.

Although both the study by Black et al. [32] and the BEACHES study estimated similar, relatively low health risks, this present study is important because it shows how the probability of adverse health consequences associated with children’s play at the beach can be impacted by considering different factors related to exposure. These factors may relate to the hazard itself, the source of the hazard, and/or the exposure route. When considering children’s potential skin contact with sand given their type of play behaviors at the beach, for example, health risks may increase by orders of magnitude as shown for benzo[b]fluoranthene in tar and assuming the dermal exposure route (Table 4). Thus, more refined health risk assessments that consider the ways children play in the beach environment can identify specific exposure factors—such as the type of recreational behavior on the beach that impacts the extent of contact with (contaminated) sand, or duration and frequency of beach visits—that drive children’s health risk. The risk assessment process in the BEACHES study demonstrates how complex exposure-related information can be distilled to better measure the ways environmental hazards pose risks to children.

This comparative analysis shows that the estimated cancer risks are sensitive to population-specific factors, particularly for exposure frequency. The study of children’s environmental health is often focused on assessing health consequences associated with inhalation exposure in outdoor environments, where children engage in physical activity [46]. However, the combination of crawling, non-dietary ingestion, and potential contamination of beach sand broaden the scope of potential health risks to children playing on a beach [21,47]. Since existing oil spill chemical concentration data are limited, future risk assessments using BEACHES behavior and exposure information should incorporate chemical concentration data from oil spill trajectory simulations. This integrative risk assessment will provide information for public health agencies to better communicate risk to families and communities following an oil spill.

## 5. Conclusions

The application of exposure-related factors collected in the BEACHES study resulted in cancer risk estimates being lower for oral and dermal exposures than the previous assessment using default parameter values [32], yet higher for inhalation (assuming inhalation is from suspended solid-phase particulates). However, a Monte Carlo simulation resulted in the mean cancer risk estimations for oral and dermal exposures to be similar to risk estimates from the previous risk assessment for the same exposure routes. When considering both studies, dermal-related risks were higher than the other exposure routes considered. Future assessments should consider different beach geographies to evaluate how sand particle size can influence adherence to the skin and, therefore, dermal exposure. Exposure-related parameters typically contribute the greatest variability and uncertainty in risk assessments. Additional behavior-related information obtained from the translation of BEACHES videotaped data will inform a more refined risk assessment, including comparing risks by age, gender, beach location, and other exposure factors and exposure durations with various media (e.g., sand and water).

## Figures and Tables

**Table 1:** Chemical-specific factors for oral, dermal, and inhalation exposure routes. Data are consistent with factors used in risk analysis by Black et al. [32]. Factors were originally reported by the Center for Environmental and Human Toxicology (CEHT) [41].

	Oral		Dermal		Inhalation
Factor	Relative Bioavailability Factor, *RBA* (Fraction)	Oral Slope Factor (kg∗Day/mg)	Absorption Factor, ABS (Unitless)	Dermal Slope Factor (kg∗Day/mg)	Inhalation Slope Factor (kg∗Day/mg)
**Benzo[b]fluoranthene**	0.5	0.73	0.01	1.46	0.31

**Table 2:** Inputs for Monte Carlo analysis using Crystal Ball. Type of data distribution, minimum, maximum, and likeliest values were inputted for each dataset [21].

	ASSUMPTIONS			
	Distribution	Minimum	Likeliest (Median)	Maximum
EXPOSURE DATA				
**Body Weight (kg)**	Triangular	19.2	34.8	82.4
**Skin Surface Area (cm^2^)**	Triangular	7430	12,582	21,258
**Frequency of Exposure (days/year)**	Triangular	1	3	50
CHEMICAL DATA				
**Benzo[b]fluoranthene (mg/kg)**	Triangular	0.62	1.46	4.40

**Table 3:** Population-specific factors that were assumed by Black et al. [32]. Average values were calculated for body weight, frequency of exposure, and skin surface area from Beach Exposure and Child Health Study (BEACHES) study data.

Factor	Risk Assessment Variables (Black et al., 2016) [32]	Risk Assessment Variables from BEACHES Study
**ALL PATHWAYS**		
Body Weight, BW (kg)	25.4	34.8
Frequency of Exposure, F (days/year)	12	3
Exposure Duration, ED (years)	8	
Average Time, AT (days)	365 (non-cancer) 28,489 (cancer)	
Exposure Factor, EF (unitless)	0.263 (non-cancer) 0.002948 (cancer)	0.0658 0.000842
**ORAL**		
Soil Intake Rate, IR_S_ (mg/day)	1000	
Conversion Factor, CF (mg/kg)	0.000001	
**DERMAL**		
Skin Surface Area, SA (cm^2^/event)	11,350	12,582
Adherence Factor, AD, AF (mg/cm^2^)	18	
Conversion Factor, CD, CF (mg/kg)	0.000001	
**INHALATION**		
Soil-to-Air Particulate Emission Factor, PEF (m^3^/kg)	1,240,000,000	
Inhalation Rate, IR_a_ (m^3^/day)	9.62	
Exposure Time, ET (hours/day)	3	

**Table 4:** Cancer risk estimates for benzo[b]fluoranthene associated with oral, dermal, and inhalation exposure from concentrations found in sediment, weathered oil, and tar for both Black et al. [32] (assuming default exposure parameters) and the BEACHES study (applying exposure information obtained from the BEACHES study).

Cancer Risk—Benzo[b]fluoranthene				
	EXPOSURE ROUTE			
	ORAL	DERMAL	INHALATION	TOTAL RISK
	Black et al. BEACHES	Black et al. BEACHES	Black et al. BEACHES	Black et al. BEACHES
**Sediment**	6.20 × 10^−8^ 1.29 × 10^−8^	5.10 × 10^−7^ 1.17 × 10^−7^	4.10 × 10^−13^ 2.55 × 10^−13^	5.72 × 10^−7^ 1.30 × 10^−7^
**Weathered Oil**	1.90 × 10^−7^ 3.89 × 10^−8^	1.50 × 10^−6^ 3.52 × 10^−7^	1.20 × 10^−12^ 7.69 × 10^−13^	1.69 × 10^−6^ 3.91 × 10^−7^
**Tar**	2.60 × 10^−8^ 5.48 × 10^−9^	1.60 × 10^−10^ 4.96 × 10^−8^	1.70 × 10^−13^ 1.08 × 10^−13^	2.62 × 10^−8^ 5.51 × 10^−8^
**Total**	2.78 × 10^−7^ 5.73 × 10^−8^	2.01 × 10^−6^ 5.19 × 10^−7^	5.80 × 10^−13^ 1.13 × 10^−12^	2.29 × 10^−6^ 5.76 × 10^−7^

**Table 5:** Cancer risk ranges for benzo[b]fluoranthene using Monte Carlo analysis. Datasets for body weight, frequency of exposure, and skin surface area are from the BEACHES study.

Cancer Risk—Benzo[b]fluoranthene, Monte Carlo Analysis			
	Exposure Route		
	Oral	Dermal	Inhalation
**Mean**	2.63 × 10^−7^	2.82 × 10^−6^	5.13 × 10^−18^
**2.5%**	1.74 × 10^−8^	3.02 × 10^−7^	4.17 × 10^−19^
**97.5%**	1.51 × 10^−6^	1.70 × 10^−5^	3.02 × 10^−17^
